# Oxygen-deficient TiO_2−*x*_ interlayer enabling Li-rich Mn-based layered oxide cathodes with enhanced reversible capacity and cyclability†

**DOI:** 10.1039/d3ra02125d

**Published:** 2023-06-05

**Authors:** Yike Lei, Yingchuan Zhang, Yongkang Han, Jie Ni, Cunman Zhang, Qiangfeng Xiao

**Affiliations:** a School of Automotive Studies, Clean Energy Automotive Engineering Center, Tongji University (Jiading Campus) 4800 Cao'an Road Shanghai 201804 P. R. China xiaoqf@tongji.edu.cn

## Abstract

The unique anion redox mechanism of Li-rich Mn-based layered oxide (LMLO) cathodes endows them with a higher specific capacity compared with conventional cathodes. However, the irreversible anion redox reactions can cause structural degradation and sluggish electrochemical kinetics in the cathode, resulting in a poor electrochemical performance in the batteries. Thus, to address these issues, a single-sided conductive oxygen-deficient TiO_2−*x*_ interlayer was applied on a commercial Celgard separator as a coating layer towards the LMLO cathode. After coating TiO_2−*x*_, the initial coulombic efficiency (ICE) of the cathode increased from 92.1% to 95.8%, the capacity retention improved from 84.2% to 91.7% after 100 cycles, and the rate performance of the cathode was significantly enhanced from 91.3 mA h g^−1^ to 203.9 mA h g^−1^ at 5C. Operando differential electrochemical mass spectroscopy (DEMS) showed that the coating layer could restrain the release of oxygen in the battery, especially from the initial formation process. The X-ray photoelectron spectroscopy (XPS) results demonstrated that the favorable oxygen absorption by the TiO_2−*x*_ interlayer benefitted the suppression of side reactions and cathode structural evolution and favored the formation of a uniform cathode-electrolyte interphase on the LMLO cathode. This work provides an alternative path to address the issue of oxygen release in LMLO cathodes.

## Abstract 2

The oxygen vacancies in the TiO_2−*x*_ interlayer inhibit the release of oxygen, while Li^+^ migrates to the anode, thus enhancing the reversibility of the cathode structural evolution and favoring the formation of a cathode electrolyte interface.

## Graphical abstract

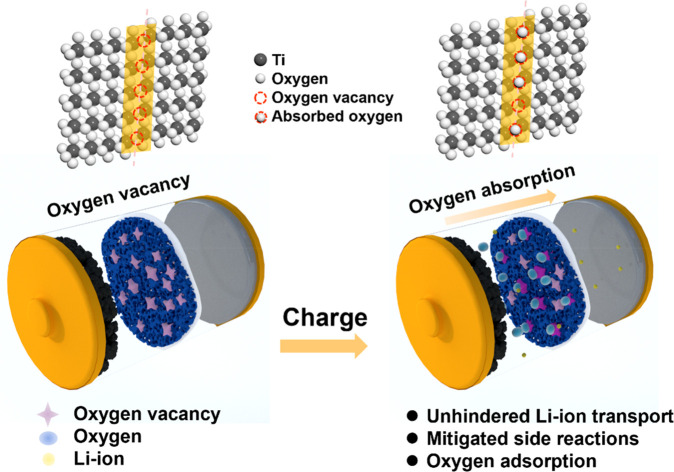

## Introduction

Lithium-ion batteries (LIBs) have gained increasing attention as efficient and clean energy storage devices to reduce the carbon dioxide emissions from fossil fuels, restraining environmental pollution,^1^ and thus the related LIB industry has developed rapidly.^2,3^ To date, LIBs have been widely applied in portable electronic devices, power tools, medical devices, smart watches, satellites, drones, marine vehicles, electric vehicles and utility-scale storage. However, the expanding applications of LIBs has also increased the battery requirements including higher energy density, longer life, lower cost, and safer features.^4^ In the current commercial LIBs, the cathodes have a much lower specific capacity than anodes and are 40% of the cost of the whole battery, which limits the development of next-generation LIBs.^5^ Benefiting from their unique anion redox mechanism,^6^ Li-rich Mn-based layered oxide (LMLO) cathodes can deliver a high specific capacity of more than 250 mA h g^−1^, and thus are considered promising cathode candidates for next-generation high-energy-density LIBs.^7^ However, LMLO cathode materials are associated with some critical issues, which should be resolved before their large-scale commercial applications, such as release of oxygen, voltage decay, and poor rate capability.^8^ Generally, the Li_2_MnO_3_ component in LMLO can be activated at 4.5 V with the concomitant release of oxygen during the first charge process.^9,10^ The release of oxygen leads to the degradation of the electrode structure from layer to spinel and aggravates the electrolyte decomposition on the surface of the electrode, resulting in poor cycle stability.^11^ In addition, oxygen and oxygen radicals generated during charge/discharge have a negative influence on the safety of batteries.^12^

Recently, many different strategies have been proposed to solve the issue of oxygen release in LMLO cathodes. For example, doping was performed to stabilize the crystal structure and reduce the release of oxygen.^13^ Also, a concentration gradient structure of LMLO was designed to increase the stability of the structure and avoid unexpected surface reactions to reduce oxygen release.^14^ In addition, various materials, such as oxides,^15^ phosphates,^16^ fluorides,^17^ lithium-ion conductors,^18^ polymers,^19^ and functionalized coatings containing oxygen vacancies, were coated on the cathode to inhibit oxygen release by reducing side reactions.^20,21^ However, doping with foreign elements and/or coating with inert compounds may reduce the capacity, and even reduce the conductivity of the material and diminish the rate capability.^21^ In addition, both methods involve complex modification processes, which possibly destroy the surface structure of the cathode materials.

Modification of the separator is also commonly employed to improve the electrochemical performance of LIBs. Although the separator interlayer inevitably reduces the energy density of the whole battery, the improvement in electrochemical performance, thermal stability and safety performance by the interlayer of separator have attracted increasing attention from researchers.^22,23^ Accordingly, researchers have investigated various coatings on the separator to improve the electrolyte wettability and thermal stability of the battery.^24,25^ Parikh *et al.* developed a binary ceramic coating consisting of Al_2_O_3_ and TiO_2_ on a thin separator to enhance the thermal stability, thermal conductivity and electrolyte wettability.^26^ Qi *et al.* proposed a mesoporous SiO_2_ (mSiO_2_)-anchored separator *via* covalent bonding, where the mSiO_2_ nanoparticles facilitated the storage of more electrolyte and enhanced the swift lithium-ion diffusion during charge/discharge.^27^ However, this type of ceramic coating layer on the separator is generally designed to enhance the electrolyte wettability, thermal stability and mechanical properties,^28^ and there is no report to date on modified separators that can store the oxygen released from LMLO during charge and discharge.

Black titanium dioxide (TiO_2−*x*_) has been widely applied in fuel cells, photoelectrochemical sensors and microwave absorbers.^29^ TiO_2−*x*_ is a member of the homologous series known as Magneli phases,^30^ where the substoichiometric titanium oxides consist of two-dimensional chains of octahedral TiO_6_, with the oxygen atom missing in every nth layer to compensate for the loss of stoichiometry.^31^ This structure ensures the excellent electrical conductivity and great corrosion resistance of TiO_2−*x*_ materials.^32,33^ In addition, TiO_2−*x*_ has good electrochemical stability in corrosive media (including organic electrolytes).^34^ Furthermore, TiO_2−*x*_ has been applied as the separator coating layer of lithium–sulfur batteries due to its high affinity for polysulfides.^35,36^ However, the use of TiO_2−*x*_ as a functional interlayer to absorb the oxygen evolved from high-energy cathodes (*e.g.*, LMLO and high-nickel cathodes) has not been reported. The evolution of oxygen is the key issue hindering the commercialization of LMLO cathode materials. As mentioned previously, oxygen evolution can cause structural degeneration, voltage decay and electrolyte decomposition, which can result in inferior electrochemical performances in LMLO.

**Fig. 1:**
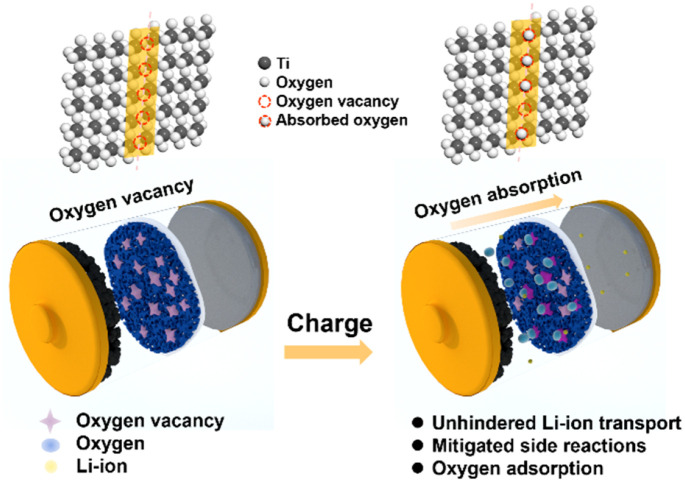
Schematic illustration of the mechanism of the TiO_2−*x*_-coated separator for the inhibition of oxygen release in LMLO cathodes by an TiO_2−*x*_ interlayer.

In this work, we proposed the use of oxygen-deficient TiO_2−*x*_ as an interlayer to suppress the oxygen release of LMLO for the first time. As shown by the schematic in Fig. 1, Li^+^ and oxygen are liberated from the cathode during the charge process and migrate to the anode. The oxygen vacancies in the TiO_2−*x*_ interlayer adsorb the released oxygen, while Li^+^ migrates to the anode. In addition, the porous structure of the coating layer on the separator facilitates the adsorption of oxygen without the hindrance of lithium-ion transportation. Consequently, this TiO_2−*x*_ interlayer improved the initial coulombic efficiency and enhanced the cyclability of the LMLO cathode due to its oxygen absorption effect and the mitigated side reactions between oxygen radicals and the electrolyte.

## Experimental

### Materials

Polypropylene (PP) separators (Celgard 2400, thickness of ∼25 μm) were purchased from Nanjing MJS Energy Technology Co. Ltd. Black titanium dioxide powder was purchased from Shanghai TaiYang Technology Co. Ltd. and milled at 200 rpm for 8 h for subsequent use. The LMLO cathode powder was obtained from Ningbo Fuli Battery Material Technology Co. Ltd. The electrolyte containing 1 M LiPF_6_ dissolved in fluoroethylene carbonate/dimethyl carbonate (FEC/DMC 1/3 by volume) was provided by Suzhou DoDoChem Technology Co., Ltd.

### Preparation of TiO_2−*x*_-coated separator

The black titanium dioxide (TiO_2−*x*_)-coated separator was prepared *via* the slurry coating method using polyvinylidene fluoride (PVDF) as the binder. Firstly, TiO_2−*x*_ powder and PVDF were mixed at a mass ratio of 9 : 1 in *N*-methyl-2-pyrrolidone (NMP) with a solid content of about 35 wt%. Then, the slurry was coated on one side of the Celgard 2400 separator *via* doctor blading and dried at 60 °C for 12 h in a vacuum drying oven. The as-prepared separators were punched into discs with a diameter of 19 mm. The TiO_2−*x*_-coated separators with different thickness were denoted as TiO_2−*x*_-1, TiO_2−*x*_-2, TiO_2−*x*_-3, and TiO_2−*x*_-4, corresponding to the TiO_2−*x*_ loading of about 0.6, 1.1, 1.5, and 2 mg cm^−2^, respectively.

### Preparation of LMLO cathode electrode

The LMLO cathode material, Super-P and PVDF were mixed at a mass ratio of 85 : 10 : 5 and dispersed in NMP solvent in a Thinky mixer. The mixed slurry was coated on carbon-coated aluminum foil using a doctor blade and dried at 80 °C in a vacuum drying oven overnight. The dried electrode was punched into discs with a diameter of 12 mm for electrochemical testing, and the active loading of each disc was about 4.5 mg.

### Electrochemical measurements

CR2032-type coin cells were assembled using a cathode electrode, separator and lithium metal in an argon-filled glove box (O_2_ < 0.5 ppm, H_2_O < 0.5 ppm). 1 M LiPF_6_ dissolved in FEC/DMC (1/3 vol%) was used as the electrolyte. The cyclability and rate capability of the coin cells were evaluated using a NEWARE workstation (NEWARE, China) at 25 °C in an incubator. For cyclability testing, the batteries were charged and discharged at 0.1C (1C = 250 mA g^−1^) for 2 cycles for the activation process, and then increased to 0.2C for subsequent cycles in the voltage range of 2–4.8 V. Electrochemical impedance spectroscopy (EIS) was carried out on an electrochemical workstation (Biological VMP3, France) in the range of 100 kHz to 10 mHz at an amplitude of 10 mV. Linear sweep voltammograms (LSV) were also investigated on the Biological VMP3 with a scan rate of 1 mV s^−1^ in the voltage range of the open circuit voltage to 5.2 V. Differential electrochemical mass spectroscopy (DEMS, Hiden Analytical, England) analysis was performed using a commercial ECC-DEMS *in situ* cell on an HPR-40 system. The cell was assembled in a glovebox. The cathode electrodes were prepared by mixing the active materials, Super-P and PVDF in the mass ratio of 85 : 10 : 5, and the total loading of the electrode was about 9 mg cm^−2^. Lithium metal was used as the counter electrode. The electrolyte was the same as that used in the coin-type cell. A Celgard 2400 separator and TiO_2−*x*_-coated Celgard 2400 separator were employed for comparison. The inert carrier gas was argon with a flow rate of 0.5 mL min^−1^. For electrochemical tests, the DEMS cell was evaluated using a LAND battery test system, which was charged and discharged at 0.1C in the voltage range of 2.0–4.8 V at 25 °C.

### Characterization

The X-ray diffraction (XRD, Rigaku Ultimate IV, Japan) patterns of the samples were collected using a powder X-ray Cu Kα radiation diffractometer (*λ* = 1.5418 Å) at a scan rate of 5° min^−1^. The morphology of the TiO_2−*x*_ powder and the separator were observed by scanning electron microscopy (SEM, Zeiss Sigma 300, Germany) and high-resolution transmission electron microscopy (HRTEM, JEM2100F, Japan). The element states of the samples were determined by X-ray photoelectron spectroscopy (XPS, Thermo Scientific ESCALAB 250Xi, USA) with an Al Kα source.

## Results and discussion

**Fig. 2:**
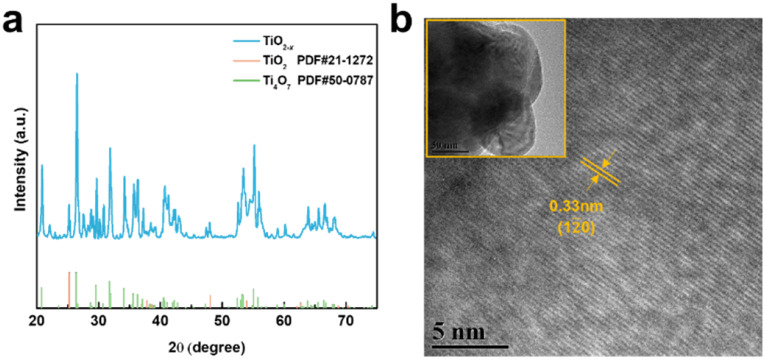
(a) XRD pattern and (b) high-resolution TEM images of TiO_2−*x*_.

**Fig. 3:**
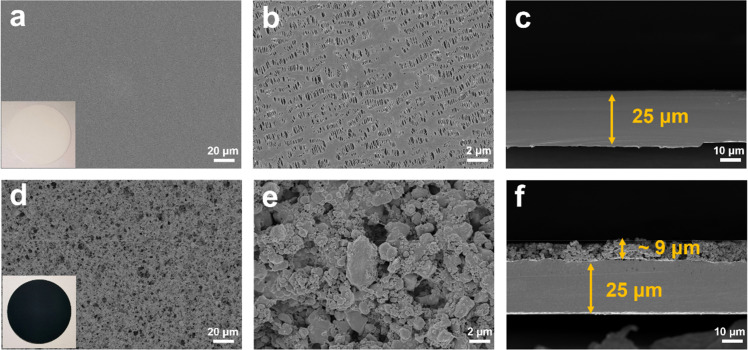
SEM images of (a–c) Celgard 2400 and (d–f) TiO_2−*x*_-coated separators.

As shown in Fig. 2a, the X-ray diffraction (XRD) patterns of the TiO_2−*x*_ powder can be mostly assigned to the standard PDF card (PDF#50-0787) of Ti_4_O_7_,^37^ which is a member of Ti_*n*_O_2*n*−1_ with super electrochemical stability and electrical conductivity. Besides, the other weak peaks are identified as that of the anatase TiO_2_ phase (PDF#21-1272). As shown by the scanning electron microscopy (SEM) images in Fig. S1a and b,† the TiO_2−*x*_ powder has an irregular shape and contains 1–2 μm particles and 200–300 nm polyhedral particles. The high-resolution transmission electron microscopy (HRTEM) images used to characterize the crystal structure of the TiO_2−*x*_ particles, as shown in Fig. 2b, demonstrate the regular spacing distance of 0.33 nm for the observed planes, which can be assigned as the (1 2̄ 0) crystal plane of the main component Ti_4_O_7_ based on their *d*-spacings.^38,39^ The top and cross-section morphology of the Celgard 2400 separator and TiO_2−*x*_-2-coated separator are demonstrated in Fig. 3. The inset images of Fig. 3a and d show that the Celgard 2400 separator is white, while the TiO_2−*x*_-coated separator is black with the TiO_2−*x*_ interlayer well attached on one side of the Celgard 2400 separator with the adhesion of the PVDF binder. As shown in Fig. 3b, the spindle-shaped holes in the Celgard 2400 separator were produced by uniaxial tensile,^27^ while the TiO_2−*x*_-coated separator was uniformly covered by TiO_2−*x*_ particles. In addition, it can be seen from the cross-section morphology that the thickness of the Celgard 2400 separator is 25 μm and the coating layer is about 9 μm, corresponding to the TiO_2−*x*_ loading of about 1.1 mg cm^−2^. The porous structure of the TiO_2−*x*_-coated separator is clearly shown in Fig. 3d–f. This porous structure in the separator provides channels for the transportation of lithium ions together with the enhanced electrical conductivity due to the favourable conductivity of TiO_2−*x*_.^34^ More importantly, the porous structure has a large surface area and can effectively improve the oxygen adsorption efficiency.

**Fig. 4:**
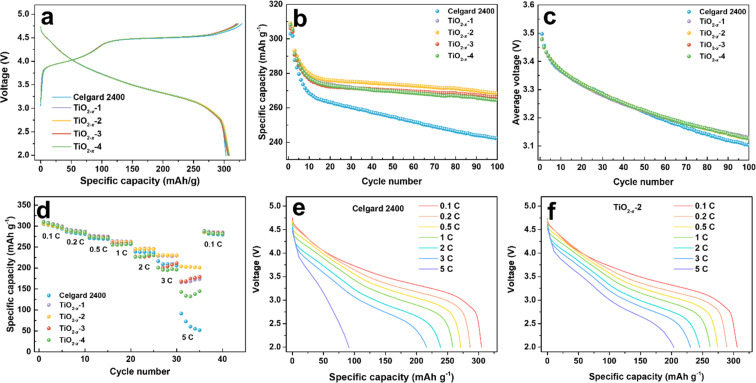
Electrochemical performance of the coin cells assembled with different separators. (a) Initial charge/charge curves at 0.1C (1C = 250 mA h g^−1^) between the voltage range of 2–4.8 V, (b) cycle performance at 0.2C after 2 cycles formation process at 0.1C, (c) average discharge voltage during cycling, (d) rate capabilities of the Celgard 2400 and TiO_2−*x*_-coated separator-assembled batteries between the voltage range of 2–4.8 V, and (e and f) corresponding discharge curves at different rates. All the coin cells are tested at 25 °C in an incubator.

To investigate the effect of the TiO_2−*x*_ interlayer on the electrochemical performance of the LMLO cathode, the coated Celgard 2400 separators with different TiO_2−*x*_ loadings were assembled with an LMLO cathode and Li-metal anode in CR2032-type coin cells. The coin cells were charged and discharged at 0.1C for the first two cycles, followed by 0.2C for cyclability testing. Fig. 4a shows the initial charge and discharge curves of the batteries with pristine and TiO_2−*x*_-coated Celgard 2400 separators. All the batteries demonstrated the typical features of LMLO materials, namely, a sloping region below 4.4 V, followed by a high voltage plateau at around 4.5 V during the first charge process. The initial coulombic efficiency (ICE) and the discharge capacity of the LMLO cathode with the pristine Celgard 2400 separator was 92.1% and 303.0 mA h g^−1^, while that for the TiO_2−*x*_-1, TiO_2−*x*_-2, TiO_2−*x*_-3 and TiO_2−*x*_-4 samples was 95.5%, 95.5%, 95.8%, and 95.3%, and their initial discharge capacities were 306.8 mA h g^−1^, 308.9 mA h g^−1^, 306.2 mA h g^−1^, and 308.2 mA h g^−1^, respectively. The ICE of the LMLO cathode with the TiO_2−*x*_-coated separators was significantly enhanced compared with the pristine Celgard 2400 separator. Moreover, the discharge capacity of the cathodes with the TiO_2−*x*_-coated separators also increased slightly compared with that with that of the pristine Celgard 2400 separator. The higher ICE and discharge capacity demonstrate the excellent reversibility of the LMLO cathode, which is due to the inhibition of oxygen release by the TiO_2−*x*_ interlayer and favorable Li-ion transport rate. In addition, the charge and discharge curves of the cathode with the pristine Celgard 2400 separator and TiO_2−*x*_ coated separators almost overlapped, which indicates that the TiO_2−*x*_-coated interlayer improved the reversibility of the electrode, while it did not participate in the lithium deintercalation and electrochemical reaction. As shown in Fig. 4b, the batteries with the TiO_2−*x*_-coated separator exhibited a similar cycling performance in the first 10 cycles as that with the pristine Celgard 2400 separator. However, with an increase in the cycle number, the discharge capacity of the battery with the pristine Celgard 2400 separator decreased rapidly. In contrast, the discharge capacity of the battery with the TiO_2−*x*_-coated separator was stable. After 100 cycles, the cycle retentions of the LMLO cathodes with the Celgard 2400, TiO_2−*x*_-1, TiO_2−*x*_-2, TiO_2−*x*_-3 and TiO_2−*x*_-4 separators were 84.2%, 90.6%, 91.7%, 91.5%, and 90.7%, respectively. In addition, the batteries with the TiO_2−*x*_-coated separator exhibited lower average voltage attenuation than that with the pristine Celgard 2400 separator. As shown in Fig. 4c, the voltage of the batteries with the coated separator similarly decreased to 3.1271 V after 100 cycles, which is higher than 3.1025 V for the battery with the pristine Celgard 2400 separator. The electrochemical performances of the batteries indicate that the separators with different TiO_2−*x*_ loadings all effectively enhanced the ICE and cycle stability of the LMLO cathode compared to the Celgard 2400 separator.

The TiO_2−*x*_-coated separator also enhanced the battery rate performance when the batteries were charged at 0.1C and discharged under different rates from 0.1C to 5C, and finally back to 0.1C. As shown in Fig. 4d, the battery with the TiO_2−*x*_-2 separator exhibited a better performance than that with the TiO_2−*x*_-1, TiO_2−*x*_-3 and TiO_2−*x*_-4 separators and delivered the specific discharge capacities of 305.9, 288.9, 274.4, 262.3, 245.5, 230.9 and 203.9 mA h g^−1^ at 0.1C, 0.2C, 0.5C, 1C, 2C, 3C and 5C rate, respectively. These results are higher than that of the battery with the pristine Celgard 2400 separator, *i.e.*, 304.8, 286.4, 271.3, 258.5, 238.8, 209.1, and 91.3 mA h g^−1^ under the same rate testing condition. When the rate returned to 0.1C, the discharge capacities mostly recovered for all the batteries. The corresponding discharge curves of the cathode with the pristine Celgard 2400 and TiO_2−*x*_-2 separators at different rates are demonstrated in Fig. 4e and f, respectively. The battery with the pristine Celgard 2400 exhibited a higher polarization and lower discharge capacity than that with the TiO_2−*x*_-2 separator. The enhanced rate performance of the battery with the TiO_2−*x*_-2 separator can be ascribed to the superior electrical conductivity of the TiO_2−*x*_ coating layer.^40^

**Fig. 5:**
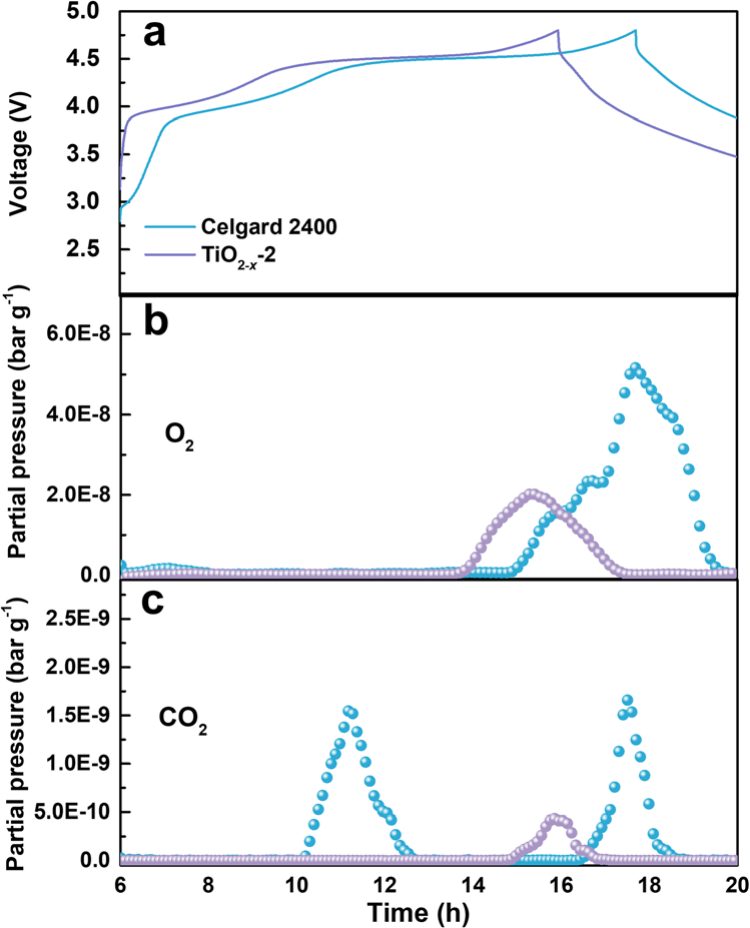
(a) First cycle voltage profiles tested in commercial ECC-DEMS cell at 0.1C with Celgard 2400 separator sample and TiO_2−*x*_-coated separator sample in the voltage range of 2–4.8 V and the corresponding (b) O_2_ and (c) CO_2_ evolution by DEMS analysis.

The gas evolution during the first charge/discharge of the cathode with different separators was analysed by operando differential electrochemical mass spectroscopy (DEMS). The effect of the TiO_2−*x*_ coating layer on the gas evolution was evaluated by analysing the evolution of O_2_ and CO_2_. The voltage curves and the gas partial pressure were examined in commercial ECC-DEMS cells, as shown in Fig. 5, where the baseline of the gas partial pressure was removed to eliminate the influence of the environment.^41^ As shown in Fig. 5b, the O_2_ evolution appeared at the end of the charge process due to the irreversible evolution of lattice oxygen, which is consistent with the literature.^42^ The release of O_2_ in the battery with the TiO_2−*x*_-2-coated separator was significantly weaker than that in the battery with the pristine Celgard 2400 separator, benefitting from the positive effect of the TiO_2−*x*_ interlayer on the oxygen absorption and reduced side reactions. The CO_2_ evolution, as shown in Fig. 5c, shows two waves for the battery with the pristine Celgard 2400 separator. The first wave of CO_2_ evolution at around 4.5 V is due to the decomposition of the alkyl carbonate solvent.^6^ The second wave of CO_2_ evolution appeared at the end of the charge, arising from the interaction between the electrolyte solvent and the LMLO lattice.^43^ Conversely, there was almost no CO_2_ wave at 4.5 V for the battery with the TiO_2−*x*_-2 separator, which may be due to the expanded electrochemical stability window induced by the TiO_2−*x*_ coating. As demonstrated by the linear sweep voltammetry (LSV) measurement of the cells containing a stainless steel working electrode and lithium foil counter/reference electrode in Fig. S2,† the electrochemical anodic stability of the electrolyte (1 M LiPF_6_ in FEC/DMC (1/3 vol%) for TiO_2−*x*_) was greatly improved by the TiO_2−*x*_ coating. In addition, the CO_2_ evolution from the battery with the TiO_2−*x*_-2 separator was significantly weaker than that from the battery with the pristine Celgard 2400 separator, which can be attributed to the mitigated side reactions between the electrolyte solvent (FEC) and LMLO lattice due to the TiO_2−*x*_ coating layer. The behaviour of O_2_ and CO_2_ evolution shows that the TiO_2−*x*_ coating layer could absorb the oxygen released from LMLO and restrain the side reactions between LMLO and the electrolyte.^44^

**Fig. 6:**
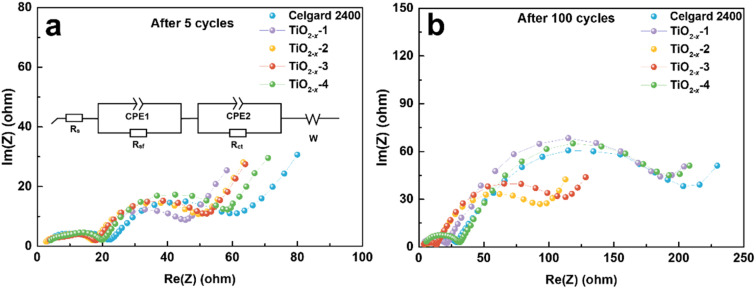
Nyquist plots measured after (a) 5 cycles and (b) 100 cycles for the coin cells used Celgard 2400 separator and TiO_2−*x*_-coated separators.

The effect of TiO_2−*x*_ coating layer on the interfacial resistance and charge transfer kinetics during battery cycling was investigated by electrochemical impedance spectroscopy (EIS). The EIS results of the LMLO‖Li batteries after 5 and 100 cycles are exhibited in Fig. 6a and b, respectively. The equivalent circuit of the Nyquist plots for the activated and cycled batteries consist of two semicircles at high and medium frequencies and a short straight line at low frequency, respectively. The high frequency semicircle is associated with the surface film resistance (*R*_sf_) and the medium frequency semicircle is associated with the charge transfer resistance (*R*_ct_). The Nyquist plots are fitted with the equivalent circuit model in the inset image in Fig. 6a and the fitting values of EIS are demonstrated in Table S1.† Obviously, the battery with the pristine Celgard 2400 separator demonstrated a higher *R*_sf_ compared with the batteries with the TiO_2−*x*_-coated separators after 5 cycles. The *R*_ct_ of the battery with the pristine Celgard 2400 separator is slightly higher than that of the batteries with the TiO_2−*x*_-coated separators. The *R*_sf_ of the battery with the pristine Celgard 2400 separator improved from 24.2 Ω after 5 cycles to 28.2 Ω after 100 cycles, which is attributed to the side reactions during the cycle process on the electrode surface. The *R*_sf_ of the batteries with the TiO_2−*x*_-2 and TiO_2−*x*_-3 separators after 100 cycles slightly decreased compared with that after five cycles, which can be ascribed to the adsorption of oxygen and inhibition of electrolyte decomposition due to the TiO_2−*x*_ coating layer during cycling. The evolution of *R*_ct_ upon cycling can explain the high stability and excellent rate performance of the battery with the TiO_2−*x*_-2 separator. The *R*_ct_ of the battery with the TiO_2−*x*_-2 separator increased from 23.8 Ω in the 5th cycle to 65.5 Ω in the 100th cycle, while the battery with the pristine Celgard 2400 separator presented a greater increase from 29.2 Ω to 174.7 Ω under the same testing condition. The value of *R*_sf_ slightly increased from 18.0 Ω to 22.3 Ω and the *R*_ct_ significantly increased from 20.2 Ω to 162.3 Ω after 100 cycles for the batteries with TiO_2−*x*_-1, which can be attributed to the insufficient inhibition of side reactions and oxygen absorption effect by the thin TiO_2−*x*_ interlayer. In the case of the battery with the TiO_2−*x*_-4 separator, the *R*_sf_ increased from 20.3 Ω to 30.0 Ω and *R*_ct_ increased from 28.7 Ω to 155.2 Ω after 100 cycles due to the thicker TiO_2−*x*_ interlayer with a porous structure, which may facilitate the absorption of more electrolyte and higher Li-ion tortuosity than the TiO_2−*x*_-2 sample. However, it is worth noting that although the total impedance values of the batteries with TiO_2−*x*_-1 and TiO_2−*x*_-4 increased significantly after 100 cycles, their impedance values were still smaller than the battery with the Celgard 2400 separator. Besides, the batteries with TiO_2−*x*_-1 and TiO_2−*x*_-4 demonstrated a higher capacity retention than the battery with the Celgard 2400 separator due to the positive effect of the TiO_2−*x*_ interlayer on the enhancement of LMLO cathode structure stability during long cycles. Combined with the O_2_ and CO_2_ evolution results, the variation in *R*_sf_ and *R*_ct_ also indicates that the TiO_2−*x*_ coating layer inhibited the release of oxygen and restrained the side reaction on the LMLO electrode.

**Fig. 7:**
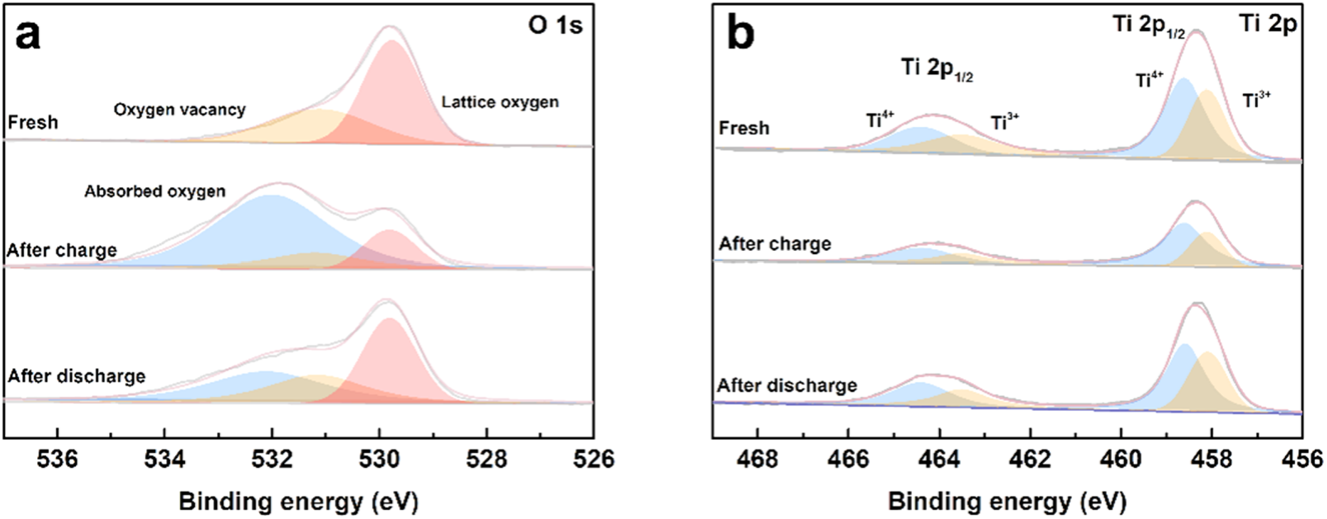
(a) O 1s and (b) Ti 2p XPS spectra of the fresh, first charged and first discharged TiO_2−*x*_-coated separators.

To evaluate the process of oxygen adsorption, the chemical states of titanium and oxygen in the TiO_2−*x*_ coating layer upon charge/discharge were detected by XPS. The TiO_2−*x*_-coated separators were obtained by disassembling coin-cells in the charged and discharged states in an Ar-filled glovebox. The O 1s and Ti 2p XPS spectra of the fresh TiO_2−*x*_-coated separator and that in the charged and discharged states from the first cycle are shown in Fig. 7a and b. As shown in Fig. 7a, the fitted peaks located at about 531.6 eV, 530.8 eV and 529.4 eV correspond to absorbed oxygen, oxygen vacancy and lattice oxygen, respectively.^45–48^ In the case of the fresh TiO_2−*x*_-coated separator, oxygen vacancies and lattice oxygen in are present in the O 1s spectra. After charging to 4.8 V, the intensity of the oxygen vacancy peak decreased and adsorbed oxygen appeared, indicating that the TiO_2−*x*_ coating layer adsorbed the oxygen released from the LMLO lattice.^49^ The intensity of the adsorbed oxygen peak decreased and the intensity of the oxygen vacancy peak increased after discharge, indicating that some of the absorbed oxygen desorbed during discharge, which may be influenced by the electric field.^50,51^Fig. 7b shows the presence of Ti^4+^ and Ti^3+^ in the Ti 2p spectra with the binding energy of Ti^3+^ lower than that of Ti^4+^, and the existence of Ti^3+^ confirms the presence of oxygen vacancies in TiO_2−*x*_.^40^ The contents of Ti^3+^ and Ti^4+^ were semi-quantitatively analysed by fitting the XPS results. The contents of Ti^3+^ for the fresh TiO_2−*x*_-coated separator and that in the charged and discharged states were determined to be 42.76%, 34.23%, and 41.28%, respectively. The excess electrons produced by the existence of oxygen vacancies could freely hop at room temperature to balance the valence states.^52–54^ These results demonstrate that the content of Ti^3+^ decreased during the battery charging process, and subsequently increased during the discharging process, which correspond to the changes in absorbed oxygen and oxygen vacancies. In addition, powder XRD was performed to investigate the possible phase changes in the crystal structure of the TiO_2−*x*_ coating layer on the separator during the charge and discharge process. As shown in Fig. S3,† the XRD patterns of the fresh TiO_2−*x*_-coated separator and that in the charged and discharged states are very similar, suggesting that there are no great or slight changes in the crystal structure of the TiO_2−*x*_ coating layer during charging and discharging.

**Fig. 8:**
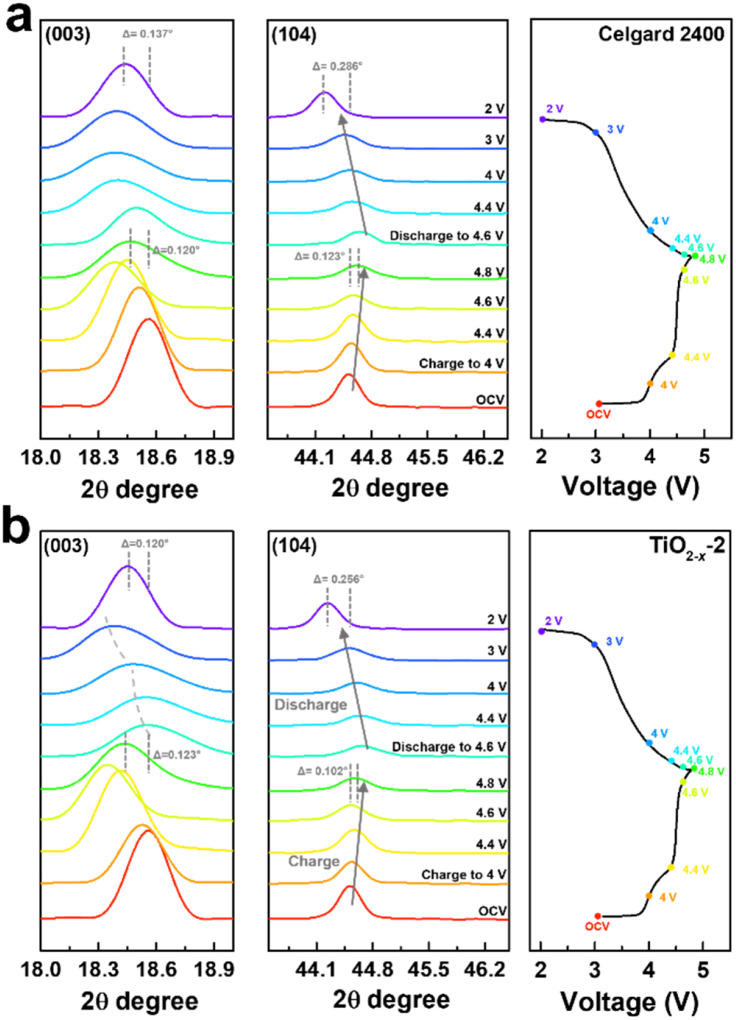
Magnified images of the (003) and (104) diffraction peaks in the XRD pattern under different charge/discharge potentials and their corresponding charge/discharge curves at 0.1C of the LMLO cathode with (a) Celgard 2400 separator and (b) TiO_2−*x*_-2 separator.

To deeply understand the structural evolution during the first charge/discharge process of the LMLO cathode and the influence of the TiO_2−*x*_ interlayer on the ICE of the LMLO cathode, *ex situ* XRD was conducted on the LMLO cathodes disassembled from the cells with a Celgard 2400 separator and with TiO_2−*x*_-coated separator at different charge/discharge potentials.^55^ The *ex situ* XRD patterns of the two samples between 10° to 45° during the first charge and discharge process are shown in Fig. S4.†^56^ As shown in Fig. 8a and b, the (003) and (104) diffraction peaks representing the lattice parameters *c* and *a* are magnified to observe the change in lattice parameters during the first charge and discharge. The LMLO crystal is arranged by periodic Li layers or transition metal (TM) layers and oxygen layers. The (003) diffraction peak corresponds to the [001] crystal direction, *c* represents the lattice distance between the oxygen layer and Li layer or TM layer, and *a* represents the lattice parameter of the crystal plane composed of lithium, TM and oxygen.^57^ During the first charge process, the (003) peak shifted to a lower angle before the charge plateau due to the removal of lithium and the increased electrostatic repulsion between the oxygen layers. Then, the (003) peak shifted to a higher angle during the charge plateau due to the activated Li_2_MnO_3_ and release of oxygen.^58^ It was found that the changes in the (003) peak in the LMLO cathode with the Celgard 2400 separator and with TiO_2−*x*_-coated separator during the first charge process are similar. During the first discharge process, similar to previous reports in the literature, the lithium ions moved to the opposite direction, the (003) peak moved to a higher angle firstly, and then to a lower angle, which is contrary to the trend during the charge process.^57^ However, it can be distinguished by comparing the two samples that the (003) peaks of the LMLO cathode with TiO_2−*x*_ separator exhibited a greater shift to a lower angle than that of the LMLO cathode with the Celgard 2400 separator, especially at a low potential before the end of the discharge. These results are probably caused by the higher reversible anion redox due to the TiO_2−*x*_ interlayer,^59^ leading to extra capacity in the low potential region (Fig. 4a).^60^ In addition, the (104) peak shifted to a higher angle during the first charge process, and then moved to a lower angle during the first discharge process, which is related to the extraction/insertion of lithium ions and the migration of TM ions.^57^ The LMLO cathode with the TiO_2−*x*_-coated separator had a smaller angle shift of the (003) and (104) diffraction peaks at the end of discharge than the LMLO cathode with the Celgard 2400 separator due to the improved reversibility during the first charge and discharge.^61^

To understand the influence of the TiO_2−*x*_ interlayer on the structural evolution of the LMLO cathode during the cycling process, the coin cells after 100 cycles were disassembled to compare the XRD patterns of the LMLO cathode with the Celgard 2400 separator and that with the TiO_2−*x*_-coated separator. As shown in Fig. S5,† the crystal structure of both LMLO cathodes degraded compared with the fresh LMLO electrode. The split peaks of (006)/(102) demonstrate the ordered layered structure, and the lower intensity ratio of (003) and (104) exhibit the higher cation mixing.^21^ The careful observation of *I*_(003)_/*I*_(104)_ shows that the cathode with the TiO_2−*x*_-coated separator it matched the fresh LMLO cathode, but was higher than the cathode with the Celgard 2400 separator. In addition, the split peaks of (006)/(102) in the LMLO cathode with the TiO_2−*x*_-coated separator were noticeable, while that in the LMLO cathode with the Celgard 2400 separator was almost indistinguishable, which may be caused by the devastating release of oxygen.^62^ These results indicate that the TiO_2−*x*_-coated separator mitigated the release of oxygen and improved the stability of the LMLO crystal structure compared to the cathode with the Celgard 2400 separator.

**Fig. 9:**
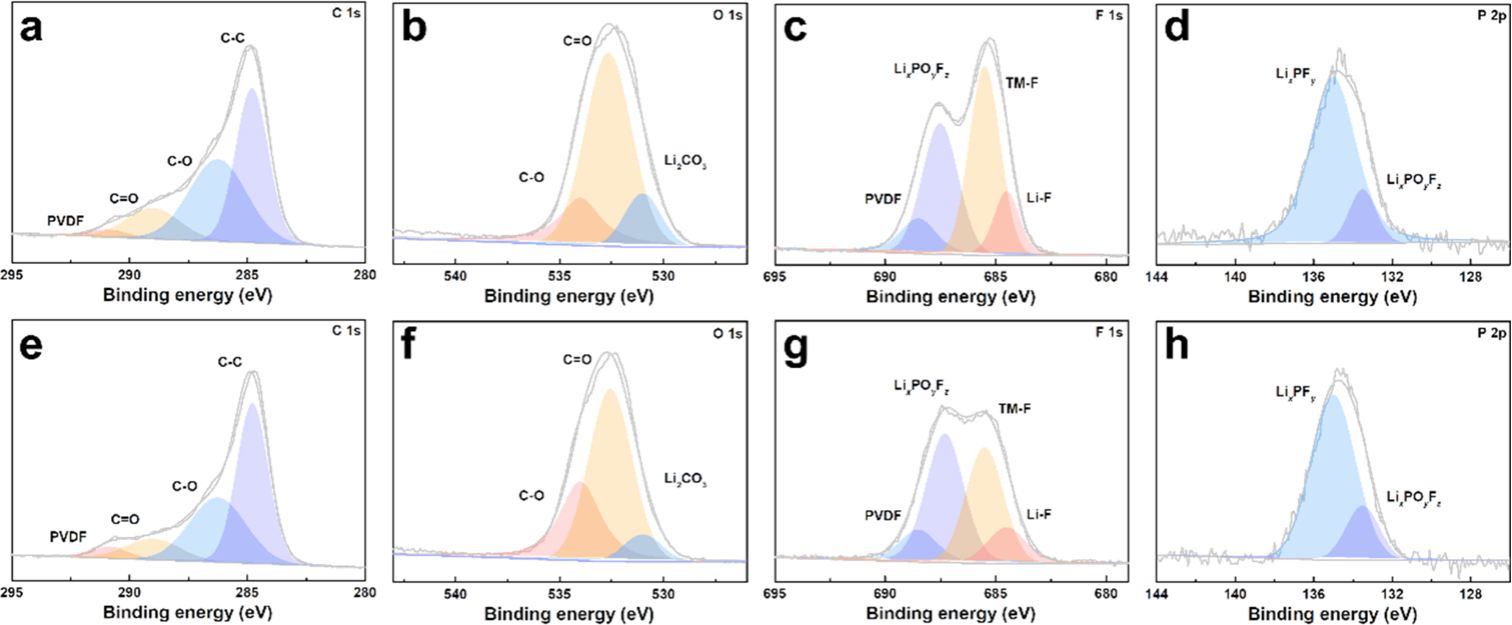
XPS spectra of LMLO cathodes after 100 cycles: (a) C 1s, (b) O 1s, (c) F 1s, and (d) P 2p for the cathode with the Celgard 2400 separator and (e) C 1s, (f) O 1s, (g) F 1s, (h) P 2p for the cathode with the TiO_2−*x*_-2 separator.

**Fig. 10:**
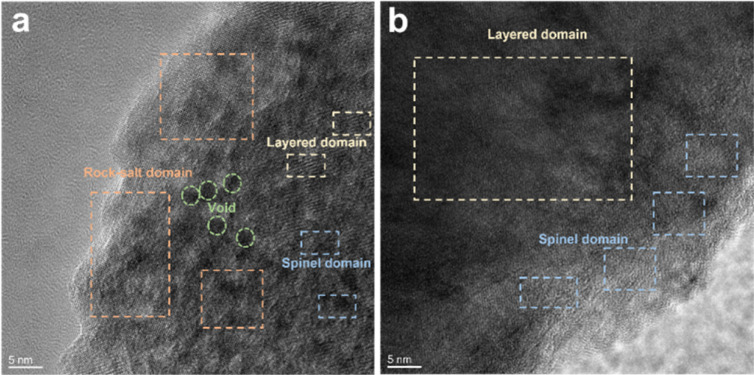
TEM images of LMLO cathodes after 100 cycles: (a) cathode with Celgard 2400 separator and (b) cathode with TiO_2−*x*_-2 separator.

The cycled cathodes with different separators were characterized by XPS to analyse the influence of the TiO_2−*x*_ interlayer on the CEI of the LMLO cathodes. The fitted peaks in C 1s (Fig. 9a and e) and O 1s (Fig. 9b and f) of C=O and C–O are attributed to the decomposition of carbonate and side reactions. By comparing the two samples, there were more C=O and C–O species on the cathode with the Celgard 2400 separator than the cathode with the TiO_2−*x*_-2 separator. In addition, the cathode with the Celgard 2400 separator demonstrated more Li_2_CO_3_ on its surface according to the O 1s spectra, which is harmful to the cathode.^63^ According to the F 1s spectra in Fig. 9c and g and P 2p spectra in Fig. 9d and h, both samples contained Li_*x*_PF_*y*_ and Li_*x*_PO_*y*_F_*z*_, which is attributed to the decomposition of LiPF_6_ during cycling.^21^ However, the cathode with the Celgard 2400 separator exhibited more TM–F and Li–F species, which may be derived from the strong reactions between the LMLO lattice and the electrolyte. To demonstrate the influence of the TiO_2−*x*_ interlayer on the CEI morphology and structure decay of the LMLO cathode intuitively, the HRTEM images of the cathode after 100 cycles are shown in Fig. S6† and 10. As shown in Fig. S6,† the LMLO cathode with the Celgard 2400 separator exhibited a discontinuous and nonuniform CEI, while the LMLO cathode with the TiO_2−*x*_-2 separator exhibited a continuous CEI with a thickness of about 4 nm, which is thinner and more uniform than that on the LMLO cathode with the Celgard 2400 separator. In addition, Fig. 10 demonstrates the structural decay of the cycled cathodes with the Celgard 2400 and TiO_2−*x*_-2 separators. Fig. 10a shows that there are plenty rock-salt structure domains in the surface region, and many voids and spinel-like structures in the inner region together with a few layered structures. In contrast, as shown in Fig. 10b, spinel-like structure domains appeared near the surface and the layered structure was well-maintained in the inner regions due to the absorption of oxygen and inhibition of side reactions by the TiO_2−*x*_ interlayer.

Considering the potential effect of the electrochemical shuttle on the cell components,^64^ the morphology and composition of the Li anode-faced sides of the separator may be valuable to analyze the effect of the TiO_2−*x*_ interlayer and cathode stability.^65^ Fig. S7a† shows that a part of the SEI remained on the surface of the Celgard 2400 separator with large broken Li dendrites embedded in the separator, while the surface of the Li anode-faced side of the TiO_2−*x*_-coated separator (Fig. S7b†) appeared smoother and had a much smaller quantity of particles. In the XPS spectra shown in Fig. S8,† the products of carbonate decomposition and Li oxide were observed on the Li anode-faced side Celgard 2400 separator, while many Li fluoride components were observed on the Li anode-faced side TiO_2−*x*_-2 separator. The aforementioned results further indicate that the TiO_2−*x*_ coating layer on the separator prevented the occurrence of excessive side reactions and enhanced the cathode stability, which can be attributed to the absorption of oxygen on the TiO_2−*x*_ coating layer, preventing oxygen and oxygen radicals from crossing the separator and enhancing the electrochemical stability at a high voltage. To determine the influence of the TiO_2−*x*_ coating layer on the impedance of the cathode and anode electrodes, a three-electrode assembly was used to demonstrate the resistance of the LMLO cathode and Li anode separately.^66^ As shown in Fig. S9,† the impedance of the three-electrode was roughly consistent with the EIS of the two-electrode coin-cell configuration. In the initial cycles, the impedance values of the Li anode were almost the same, while the impedance values of the LMLO cathode continued to change with an increase in the cycle numbers. This result indicates that the TiO_2−*x*_ interlayer mainly benefitted the formation of a more conductive CEI at cathode side.

## Conclusion

A uniform oxygen-deficient TiO_2−*x*_ interlayer with controllable thickness was applied to the surface of a commercial Celgard separator for high-performance LMLO cathodes. Compared with the pristine separator, the TiO_2−*x*_ interlayer significantly enhanced the electrochemical performance of the LMLO cathodes. The initial coulomb efficiency, cycle retention after 100 cycles, and capacity at 5C of the LMLO cathodes increased from 92.1%, 84.2% and 91.3 mA h g^−1^ to 95.8%, 91.7%, and 203.9 mA h g^−1^, respectively. This performance is ascribed to the inhibition of oxygen release and enhancement of electrochemical stability by the TiO_2−*x*_ coating layer, which mitigated the side reaction between LMLO and the electrolyte, enhanced the reversibility and inhibited the structural decay of the LMLO electrode. In addition, the TiO_2−*x*_ coating layer prevented oxygen from crossing the separator and favored the formation of a uniform CEI, benefiting the cyclability of the cathode electrode. This work provides a novel path to improve the performance of LMLO cathodes using an oxygen-deficient TiO_2−*x*_ interlayer.

## Conflicts of interest

There are no conflicts to declare.

## Supplementary Material

RA-013-D3RA02125D-s001
