# Supplementary material for: Oxygen-deficient TiO2−x interlayer enabling Li-rich Mn-based layered oxide cathodes with enhanced reversible capacity and cyclability
Source: RSC Adv. 2023 Jun 5;13(25):16850–9. doi: 10.1039/d3ra02125d (PMC10240256; doi:10.1039/d3ra02125d)
Supplement: RA-013-D3RA02125D-s001 [file RA-013-D3RA02125D-s001.pdf]

Electronic Supplementary Material (ESI) for RSC Advances.
This journal is © The Royal Society of Chemistry 2023

Supporting Information

Oxygen-deficient TiO_{2-x} interlayer enabling Li-rich Mn-based layered oxide cathodes with enhanced reversible capacity and cyclability

Yike Lei, Yingchuan Zhang, Yongkang Han, Jie Ni, Cunman Zhang, and Qiangfeng Xiao*

School of Automotive Studies & Clean Energy Automotive Engineering Center, Tongji University (Jiading Campus), 4800 Cao'an Road, Shanghai 201804, P. R. China

*Corresponding author: xiaoqf@tongji.edu.cn (Q. Xiao)

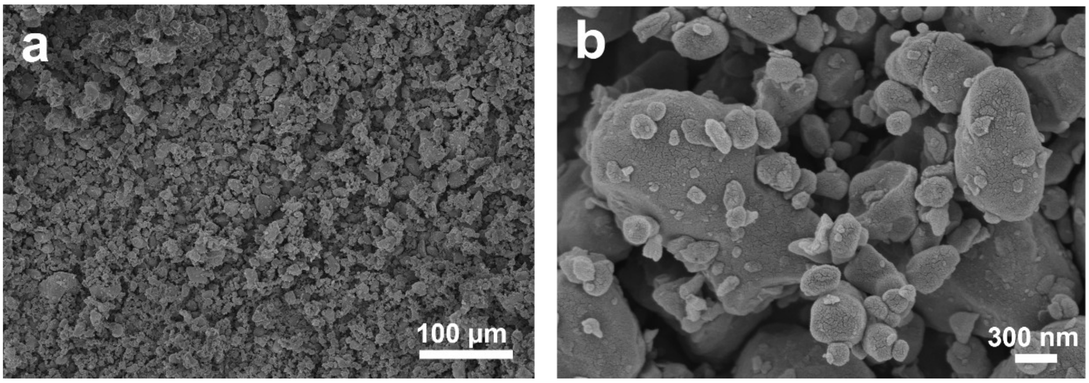

Fig. S1. The SEM images of the TiO_{2-x} powder.

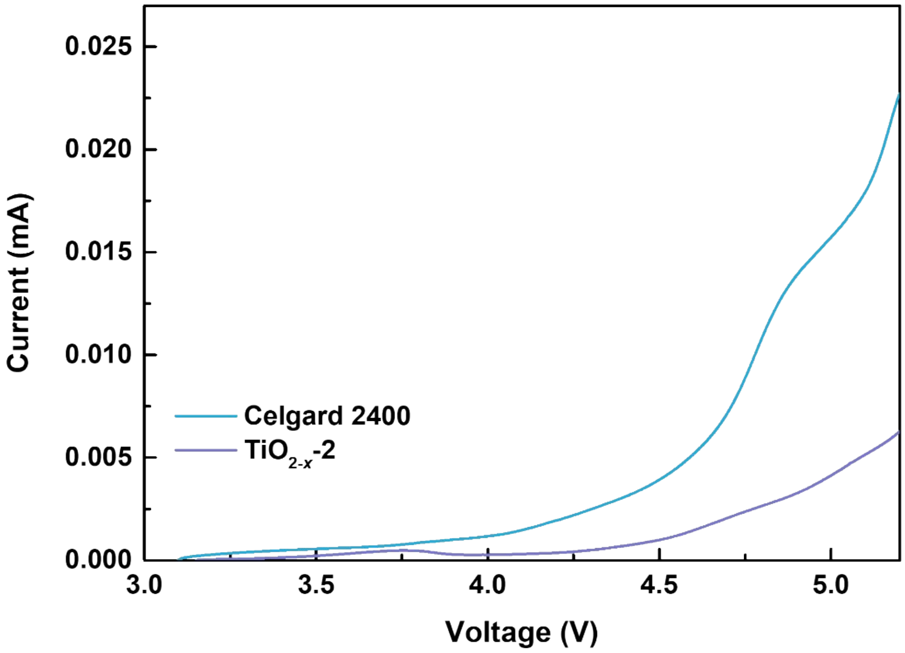

Fig. S2. Linear sweep voltammetry (LSV) curves of Celgard 2400 and $TiO_{2\text{-}x}$-2 separator between open circuit voltage and 5.2 V.

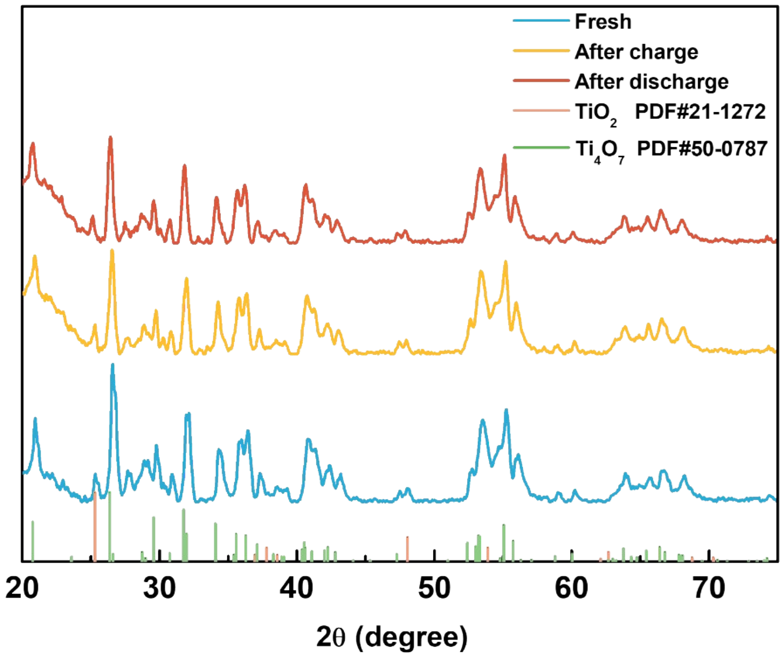

Fig. S3. XRD pattern of the uncycled separator, first charged and first discharged $TiO_{2\text{-}x}$ coated separator.

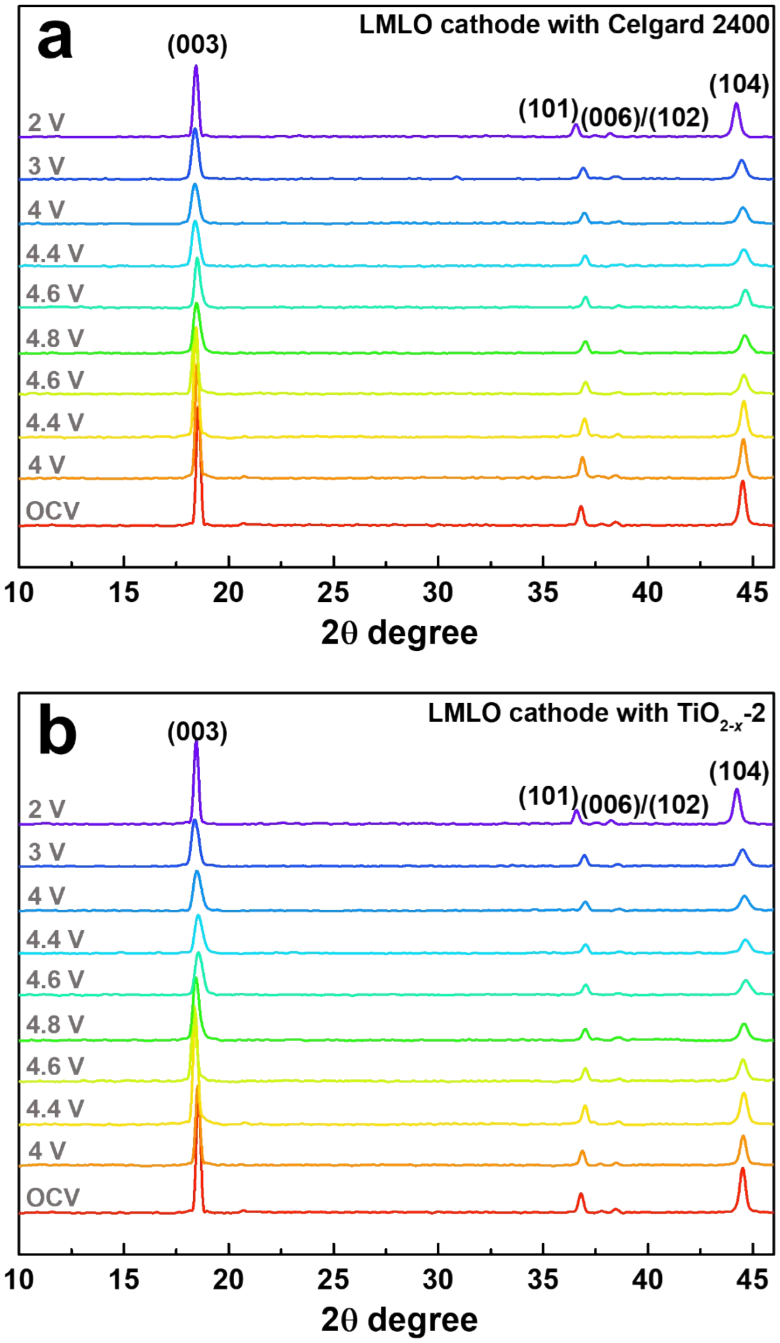

Fig. S4. The ex-situ XRD patterns of the LMLO cathode with (a) Celgard 2400 and (b) TiO_{2-x}-2 between 10° to 45° during first charge/discharge process under different charge/discharge potentials.

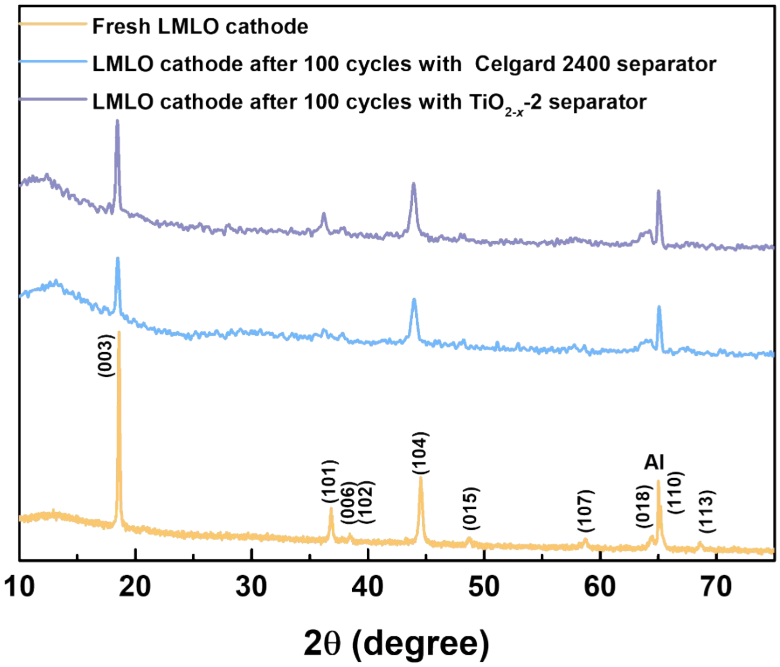

Fig. S5. The XRD patterns of fresh electrode, LMLO cathode after 100 cycles with Celgard 2400 and $TiO_{2\text{-}x}$-2 separator.

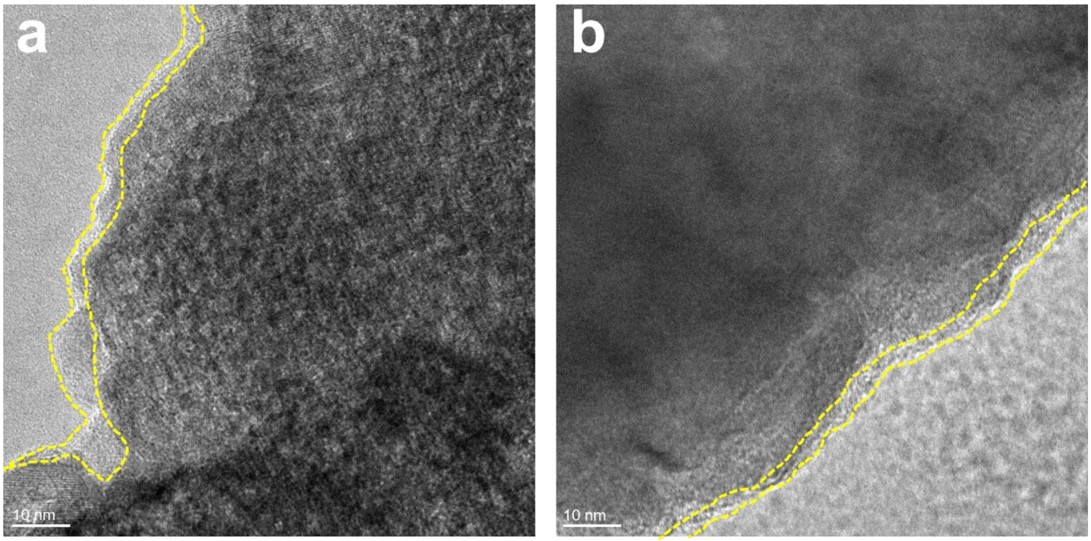

Fig. S6. The morphology of CEI in LMLO cathodes which after 100 cycles: (a) the cathode with Celgard 2400 separator and (b) the cathode with $TiO_{2\text{-}x}$-2 separator.

Since the organic components on the surface will disappear when the electron beam is focused, two images were selected to show thee CEI morphology and the structural decay.

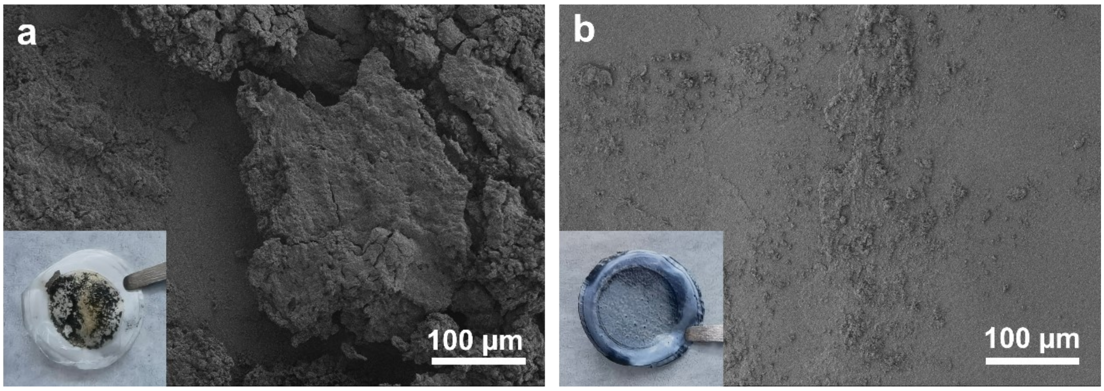

Fig. S7. SEM images and the inset photograph on the Li anode-faced side of the separators which after 100 cycles for (a) Celgard 2400 and (b) TiO_{2-x}-2 separator.

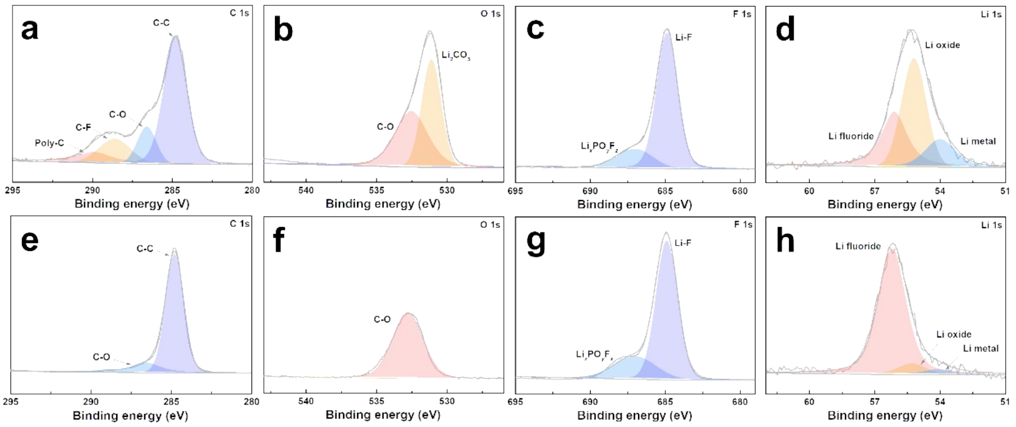

Fig. S8. XPS spectra of Li anode-faced sides separators which after 100 cycles: (a) C 1s, (b) O 1s, (c) F 1s, (d) Li 1s for Celgard 2400 separator and (e) C 1s, (f) O 1s, (g) F 1s, (h) Li 1s for TiO_{2-x}-2 separator.

The Li anode-faced sides of the celgard 2400 and TiO_{2-x} coated separators were also analysed by XPS. Fig. S8a shows the C 1s results of the Celgard 2400 separator sample, the fitted peaks of poly C, C-F and C-O are attributed to the carbonate decomposition and side reactions. In contrast, there is only a little C-O by-products on the TiO_{2-x}-2 separator sample from the C 1s fitted results in Fig. S8e. Regarding the O 1s spectra, there are TM-O on the Celgard 2400 separator sample in Fig. S8b, caused by the dissolution and migration of transition metal ions from the LMLO cathode. Note that no TM-O has been observed in the TiO_{2-x}-2 separator sample in Fig. S8f. From F 1s spectra in Fig. S8c and g, both samples present a dominant lithium fluoride phase as well as a small amount of $Li_xPF_yO_z$. The fitted peaks of Li 1s in Fig. S8d show that there are lithium fluoride, lithium oxide and lithium metal species for the Celgard 2400 separator sample while those in Fig. S8h are mainly ascribed to lithium fluoride for the TiO_{2-x}-2 separator sample, which can benefit the cyclability of rechargeable Li metal anode.

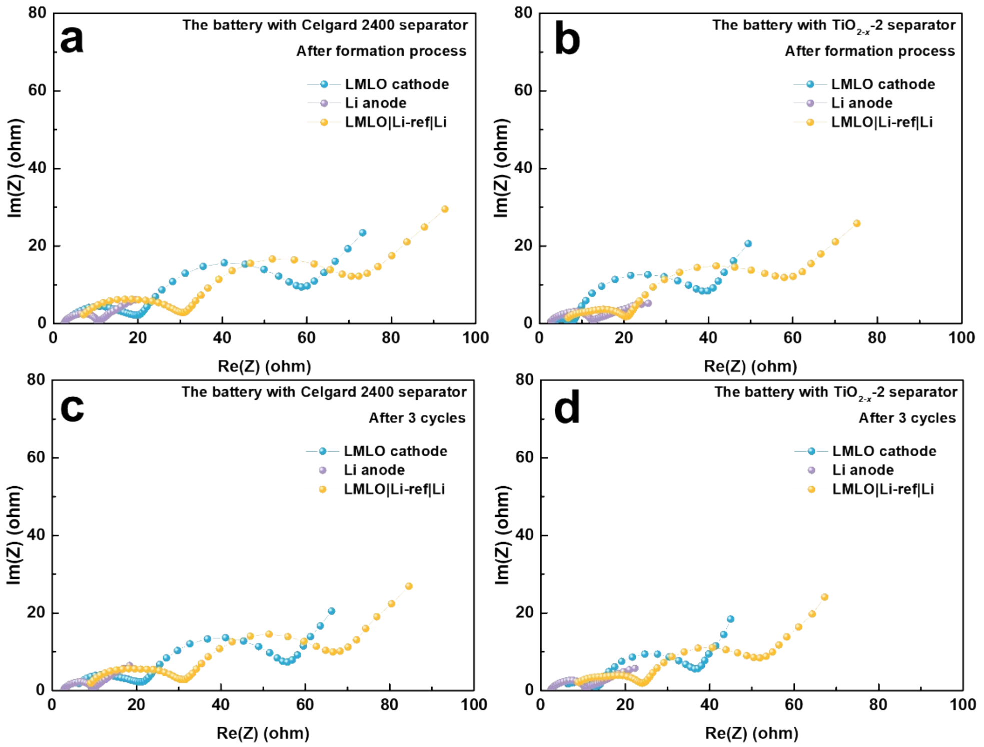

Fig. S9. Nyquist plots measured after (a) and (b) formation process, (c) and (d) 3 cycles for the three-electrode coin cells used Celgard 2400 separator and TiO_{2-x}-2 separators.

The three-electrode coin cells in this work are assembled according to the ref. 66. After the coin cell assembled, the coin cell is sealed with hot melt adhesive, and then lithium is deposited for 4 h at 0.25 mA on the Cu wire to form the reference electrode. The EIS of three-electrode coin cells are tested on BioLogic VMP3 system within the frequency of 10^{-2}–10^{5} Hz.

Table S1. Impedance parameters fitted by the equivalent circuit of the batteries with Celgard 2400 and TiO_{2-x} coated separators after 5 cycles and 100 cycles.

		**Celgard 2400**	**TiO_{2-x}-1**	**TiO_{2-x}-2**	**TiO_{2-x}-3**	**TiO_{2-x}-4**
After 5 cycles	R_s	2.5	1.9	1.6	2.4	1.6
	R_{sf}	24.2	18.0	18.0	19.8	20.3
	R_{ct}	29.2	20.2	23.8	27.1	28.7
After 100 cycles	R_s	1.9	1.5	1.3	1.3	2.3
	R_{sf}	28.2	22.3	14.8	14.7	30.0
	R_{ct}	174.7	162.3	65.5	83.3	155.2
